# Metabolic changes in concussed American football players during the acute and chronic post-injury phases

**DOI:** 10.1186/1471-2377-11-105

**Published:** 2011-08-23

**Authors:** Luke C Henry, Sébastien Tremblay, Suzanne Leclerc, Abdesselam Khiat, Yvan Boulanger, Dave Ellemberg, Maryse Lassonde

**Affiliations:** 1 Centre de Recherche en Neuropsychologie et Cognition, Department of Psychology, University of Montreal, Montréal, Québec, Canada; 2 Centre de Recherche en Neuropsychologie et Cognition, Department of Kinesiology, University of Montreal, Montréal, Québec, Canada; 3 Department of Radiology, University of Montreal and Hôpital Saint-Luc, Montréal, Québec, Canada

**Keywords:** MRI spectroscopy, sports concussion, recovery, metabolism

## Abstract

**Background:**

Despite negative neuroimaging findings many athletes display neurophysiological alterations and post-concussion symptoms that may be attributable to neurometabolic alterations.

**Methods:**

The present study investigated the effects of sports concussion on brain metabolism using ^1^H-MR Spectroscopy by comparing a group of 10 non-concussed athletes with a group of 10 concussed athletes of the same age (mean: 22.5 years) and education (mean: 16 years) within both the acute and chronic post-injury phases. All athletes were scanned 1-6 days post-concussion and again 6-months later in a 3T Siemens MRI.

**Results:**

Concussed athletes demonstrated neurometabolic impairment in prefrontal and motor (M1) cortices in the acute phase where NAA:Cr levels remained depressed relative to controls. There was some recovery observed in the chronic phase where Glu:Cr levels returned to those of control athletes; however, there was a pathological increase of m-I:Cr levels in M1 that was only present in the chronic phase.

**Conclusions:**

These results confirm cortical neurometabolic changes in the acute post-concussion phase as well as recovery and continued metabolic abnormalities in the chronic phase. The results indicate that complex pathophysiological processes differ depending on the post-injury phase and the neurometabolite in question.

## Background

The perception of sports concussions has undergone a gradual overhaul throughout the past decade where an injury that was once considered to be inconsequential has come to be understood within the neuropsychological and medical communities to be an injury with quantifiable changes to the brain that are both transient [1-3] and persistent [4-7]. According to the current literature, transient changes are by far more abundant as most of these occur within the acute phase where athletes exhibit neurocognitive changes [8-10] in addition to neurophysiological alterations [11-15]. Persistent changes have also been documented [4-7,16-19], though some doubt their clinical legitimacy, citing litigation and other secondary gains as confounds [20-23].

There is a disproportionate amount of research focusing on the acute post-injury phase owing largely to the fact that this is where the most overt effects of a sports concussion can be detected. The acute post-injury phase has no strict cut-off but is generally understood to be within three months though 80-90% of patients exhibit full recovery within the first 10 days [24,25]. Thus the chronic phase is understood to be anywhere from three months and outward post-injury in accordance with the DSM-IV-TR definition of Post-concussion Syndrome [26].

Indeed, most of the quantifiable changes associated with sports concussion are either subclinical or recovered in the acute phase [25,27]. Typically, post-concussive symptoms all but disappear within 2-3 weeks of concussion [24,28,29] with only a small percentage of cases exhibiting post-concussion effects past the acute phase [7,30-32]. Neuropsychological findings are similar, with typical neurocognitive recovery taking place on the order of days to weeks, well within the 3-month window [1,10,15,33-35] though it can take longer in some cases, particularly if the injury is not properly managed [13,36,37].

Brain imaging studies conducted across the post-concussion timeline reveal variable results contingent upon the imaging technique used [[38] for review, see [39]]. Morphological changes are difficult to characterize using technologies like CT [40,41] and MRI [41,42] regardless of proximity to injury. Even in emergency room cases where patients are scanned within 72 hours of injury, MRI did not reveal any consistent pattern of injury across patients with injuries categorized as mild [43]. However, more recent studies have found subtle changes in the white matter of mild traumatic brain injury in non sports-related patients in the acute phase [44] and well after the acute phase [45] that are otherwise undetectable with conventional imaging techniques. Given their lack of precision in detecting signs of injury in concussion, the utility of CT and MRI in characterizing sports concussion is quite limited except in the most severe cases where symptomatology is either abnormally prolonged or severe [46].

Other imaging changes have yielded strong patterns of results in the acute (fMRI) and chronic post injury phases (transcranial magnetic stimulation-TMS, event related potentials-ERPs). Functional changes have been characterized, sharing an apparent link to symptomatology [2,3,5,47,48]. Of note, all of these studies were conducted within the acute phase when athletes were still expressing elevated symptoms. That is to say, functional alterations are linked to symptomatology and these changes dissipate concurrently with self-reported symptoms and neurocognitive recovery. Because of the strong effect of functional recovery shown there are no fMRI studies investigating sports concussion in the chronic phase. Electrophysiological techniques such as TMS [4,16,17] and ERP paradigms [18,49-51] have demonstrated alterations at various time points post injury with some remarkably enduring effects well passed the acute phase, even decades after the last injury.

With the paucity of data implicating any morphological changes and the lion's share of studies reporting functional changes, sports concussions are largely understood to be a functional injury [52]. This has been demonstrated more directly by three studies that used 1H-magnetic resonance spectroscopy (MRS) to characterize the post-concussion metabolic spectra [53-55]. Consistent to these studies is a diminished level of *N*-acetylaspartate (NAA) which is thought to be indicative of reversible neuronal and/or mitochondrial dysfunction [56]. In accordance with the decrease in energy (ATP) production [57], NAA levels fall in the acute post injury phase [53,54], but can recover in injuries that do not involve substantial permanent tissue destruction [56]. Indeed, Vagnozzi and colleagues [54] demonstrated metabolic recovery of NAA levels after 30 days in singly concussed athletes. All of this strongly suggests that there is metabolic recovery after a sports concussion, at least as it concerns NAA, but can the same be said of all neurometabolites?

Previous research has suggested that monitoring NAA levels is sufficient to conclude "full cerebral metabolic recovery" [54] after injury. Such a statement is based on two assumptions. The first is that NAA is the only neurometabolite to be affected after a concussion. Our previous work [53] demonstrated a decrease in glutamate levels in primary motor cortex in the acute post injury stage. This decrease in glutamate is consistent with the hypoglycolic state that is known to occur after closed head injury [57,58]. Indeed, while there is an immediate post-impact spike in glutamate and glycolysis, the co-occuring drop in cerebral blood flow leads to an extended energy crisis owing to the lack of available calcium resulting in impaired oxidative metabolism [57]. This drop in glutamate has been shown to correlate with injury severity in humans, and has been shown to persist for 2-4 weeks [58]. Other studies have demonstrated metabolic alterations in choline following mTBI [59-62]. Given that metabolites other than NAA have been shown to be affected due to concussion, neurometabolic recovery cannot be presumed based on NAA recovery alone. The second assumption presumes that even if other neurometabolites are affected by a concussion, all neurometabolites recover at the same rate. The current study aims to investigate this notion by comparing spectra obtained within one week (2-5 days) post concussion versus spectra obtained six months after the injury in the same athletes. Two regions of interest were employed. The first region of interest, the dorsal-lateral prefrontal cortex, was chosen based on both electrophysiological [49,51,63] and fMRI studies [2,3,5,47,48] that implicate this region in the effects of sports concussion. The second region, primary motor cortex, was chosen as a region of interest based on the alterations in intracortical motor inhibition seen in concussed athletes [4,17]. Each region of interest was imaged in the left and right hemispheres for a total of four spectra per subject. Within each region of interest, three neurometabolites will be analyzed using relative quantitation methods: NAA, glutamate, and myo-Inositol. N-acetylaspartate is present at exceptionally high concentrations in the brain second only to glutamate, and is the largest peak in spectra of healthy brain tissue [56] and is thought to be a key contributor in myelin lipid formation as well as an osmoregulator [64]. Glutamate, a member of the family of biogenic amines, is the most frequently occurring neurotransmitter in the central nervous system [65,66]. The role of glutamate is different depending on the type of receptor that it binds with: at ionotropic receptors glutamate is excitatory, whereas at metabotropic receptors it is modulatory [65]. However, glutamate has a role beyond that of neurotransmitter. It is also an important neurometabolite used in the production of nitrogen which is key in the synthesis of proteins and nucleic acid, and essential to the production of other important molecules including GABA. Myo-Inositol is synthesized throughout the body, but mostly within the brain where its concentration is also the greatest [67]. It serves as a precursor molecule for inositol lipid synthesis, but also as an osmolyte. In line with previous research we hypothesized a recovery of NAA [54] and glutamate levels [58,68] in all regions of interest. However, we further hypothesized that myo-inositol levels would be increased in the chronic phase relative to the acute phase in concussed athletes in all regions of interest based on what has been demonstrated in other TBI studies [69,70].

## Methods

### Participants

All participants in this study were active players for university level intervarsity sports teams and were recruited with help from the team physician and physiotherapists. The following exclusion criteria were applied to select the athletes who took part in this study: a history of alcohol and/or substance abuse; psychiatric illness; learning disability; neurological disorders (seizure disorder, central nervous system neoplasm, or brain tumour); and TBI unrelated to contact sports. None of the athletes who participated in this study was taking psychotropic medications at the time of testing. The study was composed of one experimental group (N = 10) examined at two different time points and a control group (N = 10) composed of athletes who had no history of head injury, sports related or otherwise, also scanned at two different time points. The experimental group consisted of 10 athletes who suffered a sports concussion. They were first scanned within the 5 days of injury (mean = 81.92 hours, *SD *= 46.74 hours). The second scan took place six months after the initial scan for the concussed group (mean = 6.375, *SD *= .41) and 18 months after the initial scan for controls (mean = 18.24, *SD *= 10.29). Though there is a vast difference in time between scans, the follow-ups for control athletes should not demonstrate significant change, neurometabolites remaining relatively stable in the uninjured brain [71]. Symptoms scores were taken from both groups at each time point using the Post Concussion Symptom Scale (PCSS).

All head injuries were classified as mild, with Glasgow Coma Scale scores ranging between 13 and 15 at the time of injury. A standardized concussion-history form was administered to obtain detailed information about the number of previous concussions (if any), approximate date(s) of each concussion, descriptions of the injury(ies), nature and duration of relevant postconcussion symptoms (confusion and/or disorientation, retrograde and/or anterograde amnesia, and loss of consciousness). Concussed athletes followed the return to play protocol that was adopted after the second consensus statement on concussions in sport [72] and re-endorsed after the third international consensus statement on sports concussion [52]. In brief, the athletes followed a graded return to play beginning with complete rest, followed by light physical activity. From there, athletes progressed to sport-specific exercise and then non-contact drills before returning to game play. As is standard for return to play, athletes only advanced to the next stage of physical activity if they remained symptom free at the previous one.

### Neuroimaging

#### MR Imaging

All studies were performed at the Unité de Neuroimagerie Fonctionelle (UNF) of the Centre de Recherche de l'Institut Universitaire de Gériatrie de Montréal, using a Siemens 3-T whole-body MRI system (Siemens, Erlangen, Germany). This study was approved by the Research and Community Ethics Boards at the UNF and the Université de Montréal and done in compliance with the code of ethics as stated in the Declaration of Helsinki. All subjects gave informed consent following careful screening for MRI compatibility.

#### MR Spectroscopy

**Figure 1:**
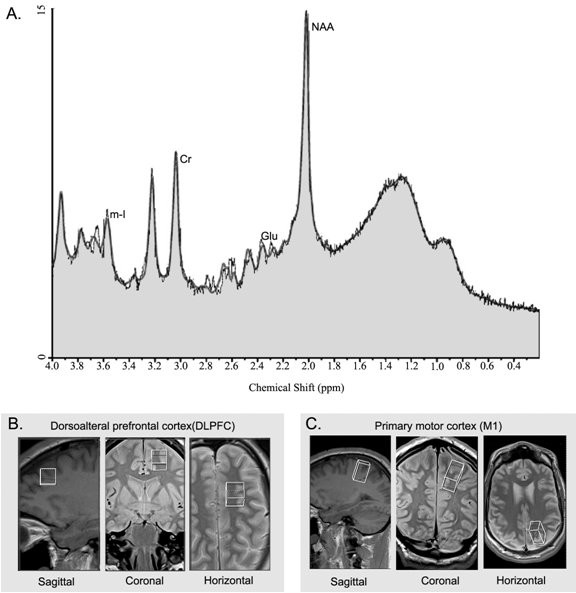
**Typical spectrum and regions of interest**. **A**. Proton spectrum of a control subject in M1 showing the peaks corresponding to the metabolites of interest, creatine (Cr), *myo*-inositol (mI), glutamate (Glu), and *N*-acetylaspartate (NAA). Concentrations are derived from the area under the peaks. **B**. Regions of interest (ROI) for MRS data acquisition depicted in the sagittal, coronal, and axial planes in the dorsolateral prefrontal cortex (DLPFC) (middle; 16 mm × 16 mm × 16 mm), and **C**. primary motor cortex (M1) (bottom; 16 mm × 20 mm × 32 mm). Spectra were recorded in both the left and right hemispheres.

We positioned our regions of interest using a rigorous anatomical localization protocol. Subjects were placed in the scanner and underwent a localizer scan prescribed parallel to the hippocampus (anterior commissure-posterior commissure). Voxels were then prescribed for the dorsolateral prefrontal cortex (DLPFC) (16 mm × 16 mm × 16 mm), and primary motor cortex (M1) (16 mm × 20 mm × 32 mm) of the left and right hemispheres (see Figure 1). All voxels were placed on an AC-PC-oriented oblique axial slice corresponding to the region of interest first on a sagittal view, and then confirmed using coronal and axial views to ensure adequate distance from ventricles, fatty tissue, and bone. Single-voxel 1H-MRS spectroscopic measurements were performed using a PRESS (Point RESolved Spectroscopy) sequence (TE (echo time) = 30 ms, TR (repetition time) = 1500 ms, 256 acquisitions, 1200 Hz bandwidth, 1024 points, duration 6:30 minutes) on a 12-channel head coil. To ensure that all four regions of interest could be captured within reasonable scan duration and to ensure the behavioural quiescence of our participants in the scanner we opted for a moderate TR and shorter TE to balance between T1- and T2-associated signal losses and scan time. Outer-volume suppression bands contiguous with the PRESS-selected volume were automatically placed in all three dimensions based on the voxel size of each ROI.

Linear Combination (LC) model (Provencher, 1993), an operator-independent spectral analysis software that estimates metabolite concentrations and their ratios relative to creatine/phosphocreatine (Cr) using a set of basis reference spectra acquired from individual metabolites on our MR instrument was used for metabolite quantitation. NAA/Cr, Glu/Cr and mI/Cr were only analyzed if the estimated uncertainties calculated as Cramer-Rao lower bounds (%SD) were less than 20%. The LCModel operator was blind to group membership.

#### Statistics

Statistical analyses were done using SPSS (PC version 16.0). Coefficients of variance (CV) were calculated for each metabolite for metabolite ratios that had overall CV values < 20% (i.e., NAA/Cr, Glu/Cr and mI/Cr). The values from the left and right hemispheres were averaged for both regions of interest in the current study. The rationale for averaging the regions of interest across hemispheres is twofold: First, the literature suggests no lateralization effects in the regions used in the current study (Zimmerman et al., 2008; King et al., 2008; Szentkuti et al., 2004; Geurts et al., 2004). Second, it is not known if the effects of a concussion are greater at the site of impact or whether any resulting changes are distributed diffusely, regardless of impact site. Because we could not otherwise be certain as to which side of the brain received the impact in the concussed athletes, we opted to combine the spectra from both hemispheres within each region of interest. The different metabolites in any given voxel are unrelated in principle and are not correlated (Braun et al. 2002). As such, the metabolite ratios of the two groups were compared using 2-way group × time repeated measures ANOVA for each metabolite in each ROI. Tests of the simple effects were carried out on metabolites in regions that differed between concussed and control athletes. Also, tests of the simple effects were also carried out on metabolites in ROIs that had been found to differ significantly between control and concussed athletes in our previous work carried out in the acute phase only [53].

## Results

Total symptom scores from the PCSS revealed a significant interaction (F (1,18) = 23.23; *p *= .000), where there was a significant effect of time (F (1,18) = 24.73; *p *= .000), and a main effect of group (F (1,18) = 5.94; *p *= .025), where concussed athletes were significantly more symptomatic in the acute post-injury phase (*p *= .003), but were statistically equivalent to controls in the chronic post-injury phase (*p *= .43) (results not shown).

**Figure 2:**
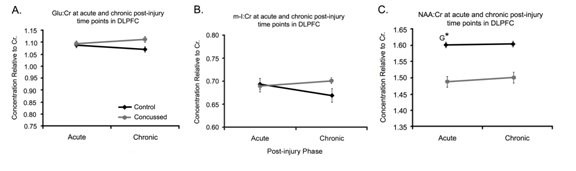
**Spectra in DLPFC**. **A**. Line graph of the mean Glu/Cr ratios, **B**. represents the means of m-I:Cr ratios and **C**. represents NAA:Cr ratios for control (black lines, n = 10) and concussed (gray bars, n = 10) athletes in the dorsolateral prefrontal cortex (DLPFC) at the acute and chronic post injury time points. Values are the mean of 24 voxel spectra (10 left hemisphere, 10 right hemisphere) per group. Standard errors of the means are represented by vertical bars. G represents a group effect and an asterisk indicates a statistically significant difference of *p *≤ 0.05.

Analyses of Glu:Cr in the DLPFC revealed no significant interaction (F (1,18) = 0.11; *p *= .75), no main effect of Time (F (1,18) = 1.23; *p *= .28) and no main effect of Group (F (1,18) = 2.28; *p *= .15) (Figure 2A). Similar results were found for m-I:Cr where there was no interaction (F (1,18) = 0.33; *p *= .58), no main effect of Time (F (1,18) = 0.02; *p *= .96), and no main effect of Group (F (1,18) = 0.72; *p *= .41) (Figure 2B). Though there was no significant interaction of NAA:Cr in DLPFC (F (1,18) = 0.69; *p *= .42) and no effect of Time (F (1,18) = 0.24; *p *= .63), there was a main effect of Group (F (1,18) = 6.87; *p *= .017). As seen in Figure 2C, the concussed group differed from the control group at both time points.

**Figure 3:**
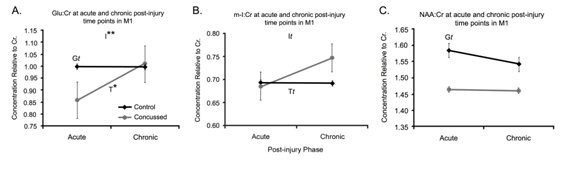
**Spectra in M1**. **A **Line graph of the mean Glu/Cr ratios, **B **represents the means of m-I:Cr ratios and **C**. represents NAA:Cr ratios for control (black lines, n = 10) and concussed (gray bars, n = 10) athletes in the dorsolateral prefrontal cortex (DLPFC) at the acute and chronic post injury time points. Values are the mean of 24 voxel spectra (10 left hemisphere, 10 right hemisphere) per group. Standard errors of the means are represented by vertical bars. I represents an interaction of Group and Time, T represents an effect of time, and G represents a group effect. Statistically, *t *represents a trend where p ≤ .10 and an asterisk indicates a statistically significant difference of *p *≤ 0.05 and a double asterisks indicates *p *≤ .01.

Within M1, Glu:Cr concentrations (Figure 3A) showed a significant time by group interaction (F (1,18) = 9.21; *p *= .007). There was also a significant main effect of Time (F (1,16) = 6.07; *p *= .024); while there was not a significant main effect of Group, there was a trend (F (1,18) = 3.48; *p *= .08). A simple effect analysis revealed a significant difference between control and concussed athletes in the acute phase (*p *= .009) but not in the chronic phase (*p *= .64). Further within group simple effects comparisons revealed no significant differences between time points for the control group (F (1,9) = 0.004; *p *= .95) while the concussed group did show a significant difference over time (F (1,9) = 11.01; *p *= .009). By contrast, m-I:Cr concentrations showed an interaction trend toward significance (F (1,18) = 2.84; *p *= .1), a trend in main effect of Time (F (1,18) = 2.79; *p *= .10) and no significant differences between Groups (F (1,18) = 2.75; *p *= .115). Despite the nonsignificance, as the group by time interaction showed p-values with a trend toward significance, further simple effects analyses were conducted (see Figure 3B). Analyses of the between-group effects revealed no significant differences in the acute phase (*p *= .93) but a significant difference in the chronic phase (*p *= .037). Within group differences between the acute and chronic phases did not reveal any significant differences in the control subjects (F (1,9) = 0.013; *p *= .91) but did reveal significant changes across time in the concussed subjects (F (1,9) = 5.23; *p *= .048). A pattern of results similar to what was shown in DLPFC was also shown in M1 concentration of NAA:Cr (see Figure 3C). There was no significant interaction (F (1,18) = 1.90; *p *= .19), nor was there a significant main effects of time (F (1,18) = 2.41; *p *= .14); however, the groups tended to differ at both time points (F (1,18) = 3.10; *p *= .095).

## Discussion

The current study investigated neurometabolic differences between 10 non-concussed athletes and 10 concussed athletes of similar age and education in the acute and chronic post-injury phases. In the DLPFC, NAA:Cr levels remained lower in the concussed group across time. All other comparisons in DLPFC revealed no significant differences or trends. Within the motor cortex there were variable changes depending upon the metabolic ratio in question. Concussed athletes demonstrated a recovery of Glu:Cr levels across time (Figure 3A). Levels of m-I/Cr were equal between control and concussed athletes in the acute post-injury phase but there was a significant difference in the chronic phase suggesting metabolic disruptions that emerged over time as opposed to being immediately reactionary to the injury. NAA/Cr levels in M1 tended to distinguish control and concussed athletes at both time points, suggesting a similar pattern as was seen in the DLPFC.

The profiles of Glu:Cr and m-I:Cr in the DLPFC demonstrate stability within and between groups. This is consistent with the findings of Shutter et al. [73] who found that Glu:Cr levels were not predictive of outcome in patients with good outcomes either immediately post-injury or eight months post-injury relative to controls. It is difficult to draw parallels with other findings involving either increases or decreases in Glu:Cr levels as other studies have taken spectra from different brain regions and in more severely injured populations [74,75]. Though we have previously demonstrated changes in Glu:Cr in primary motor cortex, we did not see similar alterations in DLPFC suggesting that there are biomechanical influences that are present in primary motor cortex that are not present in prefrontal areas [53,76]. Similarly, m-I:Cr levels were also very stable both across time and between groups.

The nature of the NAA:Cr findings was unexpected given the current literature implicating decreased levels of NAA:Cr [53,54,62,77]. Indeed, past research investigating the time course of NAA alterations report mixed results as one moves chronologically further away from the point of injury. Vagnozzi and colleagues [54] report recovery within 30 days, except for those athletes who received a second concussive blow during the acute phase. We reported a similar finding in a group of 12 athletes who were scanned days after sustaining a concussion [53]. Conversely, Cohen and colleagues [77] found that whole brain NAA levels remained depressed in patient groups that were days to over a year post injury. Other studies have found diminished NAA levels in similar brain regions ranging from days [70] to one month [62] to months [78] to a year [79] post-injury. Though our results are consistent with the latter group of studies showing continued depression of NAA:Cr levels, they are contrary to those of Vagnozzi and colleagues [54]. The exact nature of why there is continued depression is not immediately evident, but a few explanations for these metabolic alterations are plausible. Firstly, the current study's sample is composed of student athletes. Though the athletes followed the return to play protocol as specified in the consensus statements [52,72], they continued to take classes and in most cases resume practice within one week after the injury during the season in addition to continued physically demanding training in the months after the season when the follow-up data were obtained. This continued cognitive and physical effort may protract a full recovery [12,52,80] even when return to play protocols are properly followed such that a return to preconcussion levels does take place, but outside of the window used in the current study. Another possibility that may explain the continued metabolic depression is perhaps unique to contact sports like football and hockey. Even though no athletes reported a second concussion in a single season, sustaining subconcussive blows during practices and games may have also delayed metabolic recovery, even without resulting in a second injury as some studies suggest there are consequences, even if short lived, to sustaining multiple subconcussive blows [81,82]. Finally, it is also possible that sustaining a concussion persistently lowers NAA:Cr levels. There is ample evidence to suggest this is the case after a mTBI [70,77,79]. Future studies charting the time course of metabolic injury and recovery need to be conducted in order to determine whether there is recovery, in whom there is recovery, and when the recovery occurs. Though the current study investigates sports concussion, which are not necessarily equivalent to mTBI, the comparison is still worth making until more data specific to sports concussion becomes available.

Results from primary motor cortex paint a more complex picture of the metabolic state of the brain after a concussion. While depressed in the acute phase, Glu:Cr levels in concussed athletes rebound to those of control athletes in the chronic phase, elegantly demonstrating metabolic recovery. There is no precedent for Glu:Cr recovery in the mildly brain injured population, let alone in the sports literature. However, given the seemingly short lived metabolic disturbance of glutamate levels as illustrated in the neurometabolic cascade [57], we predicted just such a recovery. What remains to be further explored is when exactly between the injury and the 6-month post injury time point as measured in the current study does Glu:Cr concentration achieve physiologically typical levels and whether this metabolic resolution corresponds to symptom recovery. The reasons for affected Glu:Cr levels in M1 but not in DLPFC are not immediately apparent. However, examination of the literature investigating the biomechanics of mTBI show that the rotational forces associated with concussion suggest that M1 is consistently vulnerable to shear strain [19,76,83-85].

M-I:Cr in M1 also showed a complex pattern of results. While there are no differences in the acute phase, there appears to be a pathological increase in m-I:Cr in the chronic phase. Other studies investigating either mixed TBI groups [70] or severe TBI [69,74] though in different brain regions, have noted increased concentrations of m-I months and years after injury. The current data are consistent in this respect, but why these differences are not seen in the acute post injury phase is not immediately apparent. One possible explanation may be that there are two different mechanisms that help to regulate osmotic pressure in neurons and glia. Within the acute post-trauma phase this is primarily regulated by the rapid transport of Na^+^, K^+^, H^+^, and Cl^- ^across the plasma membrane [86,87]. Indeed, such an account is supported by the neurometabolic cascade as described by Giza and Hovda [57] where axonal swelling is indicative of hypernatremia [88]. To offset the ensuing water loss, the brain accumulates m-I to avoid a rapid over correction which could have devastating consequences to the brain [89]. Such a fast acting mechanism would preclude any observable changes in m-I in the acute phase which is in line with the current study's results. However, long term changes in cellular tonicity are offset by the transport of non-perturbing osmolytes that do not alter the electrophysiological state of the cell, namely m-I [87]. The increase in m-I might also be indicative of gliosis [see 90 for review]. Myo-Inositol increase in association with decreased NAA:Cr ratios has been associated with gliosis in other populations [90], while other work suggestions that gliosis is not necessarily related to altered neurometabolism [91]. Other studies investigating TBI also report increased m-I:Cr levels in both severe [69,74] and mild injuries [70]. In addition to TBI, other pathologies that have been associated to increased levels of m-I include drug addiction and stroke [see 67 for a complete review]. Though many questions remain as to the functional significance of such an increase in m-I, we are the first to report such an effect in the sports concussion population. Further confirmation is needed to confirm the robustness of this finding in a larger sample as well as the temporal nature of the changes.

The breadth of the metabolic changes in M1 (increases in m-I/Cr and Glu/Cr) within the concussed athletes in the chronic post-injury phase as well as the significant decrease of Glu:Cr in the acute phase may be due to the biomechanics of how the brain moves within the skull when a rotation force is applied [76,85,92]. The respective impacts of rotational and linear forces in producing a concussion [76,85,92-94] suggest that M1 is consistently vulnerable to the white matter injury of shear strain. The differential effects on Glu:Cr and m-I:Cr in M1 versus DLPFC are further corroborated by changes detected using diffusion tensor imaging where patients who had suffered a mTBI demonstrated reduced fractional anisotropy in the corticospinal tract indicating diffuse axonal injury where no such injury pattern was found in frontal regions [19].

NAA levels in M1 showed a statistical trend between concussed and control athletes. Indeed, the overwhelming evidence implicates diminished levels of NAA after a brain injury, whether it be mild [53,54,62,70,77-79,95] or severe [60,70,74,75,78,96,97]. Though the current results were not statistically significant in M1, this is consistent with what the current study demonstrates in the DLPFC. Decreased levels of NAA may be reflective of diffuse axonal injury in white matter and neuronal loss in gray matter [98], but this is a less probable interpretation given the heterogeneous nature of the neuropathological response to trauma. Declines in NAA levels are linked to decreases in ATP where the greater the initial decrease, the lesser the observed recovery. Indeed, recovery is observed in all injuries that do not include the substantial permanent destruction of brain tissue. That is to say, neurological recovery may be observed in conjunction with varying degrees of metabolic recovery where the latter need not be complete in order to observe clinical recovery in the former [56]. The persistent reduction in NAA:Cr levels observed in the current study may therefore be the consequence of a continued reduction of ATP due to the disruption of neuronal mitochondria due to the influx of Ca^2+ ^and lactate, which is consistent with the observed post-injury cellular pathology [57]; furthermore, clinical signs seem to bear little relation to the neurometabolic anomalies observed in patients suggesting a highly variable relationship between injury severity and metabolic changes past the immediate (minutes) post-injury phase [57]. It is thus conceivable, despite the findings of Vagnozzi et al (2008) that concussed athletes do have continued metabolic disruptions despite being clinically recovered in terms of their PCSS scores. That is to say, even though concussive injuries do not typically result in observable brain trauma (i.e. MRI, CT scan), and concussed individuals typically recover from a symptom standpoint within weeks after the injury, there is a sustained and persistent effect on cellular metabolism. The continued neurometabolic alterations observed in the current study may be reflective of other pathological processes such as gliosis or cell loss. Cell loss would seem to be less probable given the time frame of 6 months post injury used in the current study, though changes in volumetry have been shown as a consequence to mTBI while other studies have measured brain volume at one year post-injury [77,99]. Indeed, a study investigating mTBI 6 months post injury showed atrophy only in participants who had positive MR findings [100] while another some three months post injury also failed to find differences in participants who had suffered a MTBI [101]. Gliosis, as mentioned above is another possibility, but given that the current study observes an increase of m-I only in M1, it does not explain the persistence of metabolic disturbance observed in the DLPFC.

## Conclusions

The current study shows a complex and varying pattern of recovery and persistent metabolic depression in different cortical areas and in different metabolites. The return of Glu:Cr levels in the concussed athletes to those of controls from the acute to the chronic phase clearly demonstrates recovery, at least insofar as glutamate is concerned. The lack of group-time interactions of NAA:Cr concentration, both in M1 and DLPFC was somewhat surprising. It may be reflective of the fact that recovery from concussion is best achieved through cognitive and physical rest as described above [52,80]. It may also be reflective of a persistent pathological state. Indeed, there are several neuropathologies that demonstrate continued depression of NAA levels including, though not limited to, stroke, TBI of all severities, multiple sclerosis, brain tumours, Alzheimer disease, and neuro-AIDS and other infections [56]. Clearly these represent different pathologies operating on different mechanisms. However, it is also indicative of the global role of NAA as a marker of neurometabolic health, irrespective of the underlying pathology.

Though our results demonstrate recovery in one instance (Glu:Cr in M1), they also show continued metabolic disturbance in another (NAA:Cr in DLPFC and M1), and altogether new neurometabolic alteration in yet another (m-I:Cr in M1). While at first this may seem self-contradictory, that need not necessarily be the case. Currently, all we know about concussive neurometabolic changes is that several different neuronal processes are affected [57]. What is far less understood is how these processes are related and which of these processes are necessarily concurrent to one another. Subsequently, the recovery of these respective processes may indeed follow differential recovery curves as is already noted on a micro-level [57]. Future studies should include larger samples and more time intervals to chart the metabolic recovery and stability of not just Glu, NAA, and m-I, but also of GABA and choline containing compounds with the understanding that not all metabolites will follow the same recovery curve, nor will all brain areas.

## Declaration of competing interests

The authors declare that they have no competing interests.

## Authors' contributions

LCH was responsible for subject recruitment, data analysis and drafting of the final manuscript. ST was responsible for subject recruitment and assisted in data analysis and drafting of the manuscript. SC assisted in subject recruitment and injury diagnosis. AK and YB were responsible for spectral analyses. ED and ML participated in the design and coordination of the study and helped draft the manuscript. All authors read and approved the final manuscript.

## Pre-publication history

The pre-publication history for this paper can be accessed here:

http://www.biomedcentral.com/1471-2377/11/105/prepub
